# GSK-J4 induces cell cycle arrest and apoptosis via ER stress and the synergism between GSK-J4 and decitabine in acute myeloid leukemia KG-1a cells

**DOI:** 10.1186/s12935-020-01297-6

**Published:** 2020-06-03

**Authors:** Xuan Chu, Liang Zhong, Lihua Yu, Ling Xiong, Jian Li, Wenran Dan, Jiao Ye, Chen Liu, Xu Luo, Beizhong Liu

**Affiliations:** 1 Central Laboratory of Yongchuan Hospital, Chongqing Medical University, Chongqing, 402160 China; 2 Key Laboratory of Laboratory Medical Diagnostics, Ministry of Education, Department of Laboratory Medicine, Chongqing Medical University, Chongqing, 400016 China; 3 Clinical Laboratory of YongChuan Hospital, Chongqing Medical University, Chongqing, 402160 China

**Keywords:** GSK-J4, ER stress, Cell cycle, Cell apoptosis, PKC-α/p-bcl2 pathway, Decitabine, KG-1a cells

## Abstract

**Background:**

GSK-J4 is the inhibitor of H3K27me3 demethylase. Recent studies demonstrated that GSK-J4 could affect the proliferation and apoptosis of a variety of cancer cells. However, the effects and underlying mechanisms of GSK-J4 on the proliferation and apoptosis of human acute myeloid leukemia (AML) KG-1a cells have not been explored thoroughly.

**Methods:**

The effect of GSK-J4 on cell proliferation was assessed with CCK8, while cell cycle distribution and apoptosis were analyzed using flow cytometry. The proteins related to cell cycle, cell apoptosis, endoplastic reticulum (ER) stress and PKC-α/p-Bcl2 pathway were detected by Western blotting. The expression level of PKC-α mRNA was measured by quantitative real-time PCR.ER stress inhibitor 4-phenyl butyric acid (4-PBA) was used to explore the role of ER stress in GSK-J4 induced cell-cycle arrest and cell apoptosis. The combination effects of Decitabine and GSK-J4 on KG-1a cells proliferation and apoptosis were also evaluated by CCK8, flow cytometry and immunoblot analysis.

**Results:**

GSK-J4 reduced cell viability and arrested cell cycle progression at the S phase by decreasing the expression of CyclinD1 and CyclinA2 and increasing that of P21. Moreover, GSK-J4 enhanced the expression of apoptosis-related proteins (cle-caspase-9 and bax) and inhibited PKC-a/p-Bcl2 pathway to promote cell apoptosis. In addition, ER stress-related proteins (caspase-12, GRP78 and ATF4) were increased markedly after exposure to GSK-J4. The effects of GSK-J4 on cell cycle, apoptosis and PKC-a/p-Bcl2 pathway were attenuated after treatment with ER stress inhibitor. Furthermore, decitabine could significantly inhibit the proliferation and induce the apoptosis of KG-1a cells after combined treatment with GSK-J4.

**Conclusion:**

Taken together, this study provided evidence that ER stress could regulate the process of GSK-J4-induced cell cycle arrest, cell apoptosis and PKC-α/p-bcl2 pathway inhibition and demonstrated a potential combinatory effect of decitabine and GSK-J4 on leukemic cell proliferation and apoptosis.

## Background

Acute myeloid leukemia is a kind of severe blood disease characterized by the excessive proliferation of primitive or immature cells and inhibition of normal hematopoietic stem cells. Although some chemotherapy drugs have been used to treat AML patients, the overall survival rate is still less than 50% [1]. Therefore, the development of effective anti-leukemic drugs is necessary for the sustainable treatment of AML.

GSK-J4 is an epigenetic regulator that inhibits H3K27me3 demethylase subfamily (KDM6 subfamily members JMJD3 and UTX) and influences gene transcription by increasing total nuclear H3K27me3 levels on gene promoter [2]. Researchers have firstly discovered that GSK-J4 can reduce the production of inflammatory cytokines, such as TNF, to suppress the inflammatory reaction [3–5]. Besides, GSK-J4 can promote cell differentiation via regulating notch pathway and retinoic acid metabolism [6, 7]. In addition, it downregulates the transcription of HOX protein family, such as HOX5, HOX7 and HOX9, and then mediates cell proliferation inhibition [8]. The anti-proliferative role of GSK-J4 in reducing glutamate levels has also been identified in lung adenocarcinoma [9]. Recent investigations found that GSK-J4 could exert an inhibitory effect on various types of cancer with epigenetic dysregulation, including glioma, testicular, breast cancer and so on [10–12]. However, little is known about the role of GSK-J4 in AML and the mechanism underlying the effects of GSK-J4 on cell cycle distribution and apoptosis regulation. This motivates us to explore the potential effects of GSK-J4 on AML treatment and its underlying mechanisms.

Endoplasmic reticulum (ER) stress is a kind of pathological state that associated with the progression of a variety of diseases. When cells are exposed to different stimuli such as hypoxia, calcium metabolism disorder, oxidative stress and so on, the accumulation of unfolded or misfolded proteins can lead to ER stress. Growing evidence has suggested that early ER stress can protect cells against injuries, however, when ER was not able to deal with those stimuli, ER stress may induce apoptosis pathway [13, 14]. In addition, ER stress affects cell growth by regulating cell cycle and differentiation process [15–17]. Such roles of ER stress in cell cycle distribution and apoptosis regulation make it become an important cellular response during exposure to some anti-cancer medicine. Most studies have focused on the role of ER stress in solid tumors, yet this reaction may exert a critical effect on hematologic diseases. A number of studies have implicated that ER stress is of vital importance in the treatment of AML. For example, the combination of ER stress activator tunicamycin with conventional anti-AML drugs, such as arsenic trioxide and retinoic acid, has been found to induce cell apoptosis significantly [18, 19]. A previous study has observed that GSK-J4 can promote the differentiation of neuroblastoma cells via ER stress [20], indicating that ER stress plays a potential role in GSK-J4 treatment.

He et al. [21] demonstrated that GSK-J4 could reduce the expression of PKC-α via upregulating H3K27Me3 on PKC-α promoter and subsequently inhibiting its transcription. PKC-α is the member of PKC family, which has a strong antiapoptotic effect on cancer cells and its overexpression is closely associated with the development and progression of cancer [22, 23].

What’s more, hypomethylation drugs such as 5-azadeoxycytidine and decitabine have shown certain efficacy in the treatment of AML. Decitabine is an inhibitor of DNA methyltransferase that downregulates DNA methylation level, leading to a decrease in cell viability [24]. Recent research indicated that when combined with other drugs, decitabine could play a greater role in treating AML [25].

To date, no report had suggested that GSK-J4 can influence the proliferation, cell cycle distribution and apoptosis of AML cells via ER stress. What’s more, the relationship between ER stress and PKC-α/p-Bcl2 pathway after GSK-J4 treatment remains poorly understood. GSK-J4 is an ideal chemotherapy sensitizer when combined with other anti-cancer drugs. Yet, little is known about the synergistic effect of GSK-J4 and decitabine in AML. In this study, we took AML cell line KG-1a as research object and then explored the potential role of ER stress in GSK-J4-induced cell cycle arrest, cell apoptosis and PKC-α/p-Bcl2 pathway inhibition. Furthermore, the combination effects of GSK-J4 and decitabine on KG-1a cell proliferation and apoptosis were also evaluated.

## Materials and methods

### Reagents and chemicals

GSK-J4 (HY-15648B) and cell counting kit-8 (CCK8; HY-K0301) were purchased from MedChemExpress (NJ, USA). RPMI-1640 medium was supplied by Gibco (MA, USA), while fetal bovine serum (FBS; 900-108) was purchased from Gemini bio-products (Sacramento, CA, USA). 4-phenylbutyrate (4-PBA; 1716-12-7) were obtained from Sigma-Aldrich (St. Louis, MO, USA).

### Cell lines and culture

KG-1a cells, an AML cell line obtained from our own laboratory, were cultured in RPMI-1640 medium supplemented with 10% FBS and then incubated at 37 °C under 5% CO_2_ atmosphere for 48 h.

### siRNA transfection

The siRNA used for PKC-α and control groups were obtained from RiboBio (Guangzhou, China). For siRNA tansfection, 3 × 10^5^ cells were seeded into a 6-well plate. The specifically designed and control siRNAs were added into each well at a final concentration of 15 μM. After 48 h of transfection, the cells were harvested for apoptosis detection and Western blot analysis.

### Cell viability assay

The proliferation rates of KG-1a cells were monitored using CCK-8 assay kit. Briefly, the cells were seeded in 96-well culture plates at a density of 5 × 10^3^ cells/well. To detect the role of GSK-J4 on cell viability, KG-1a cells were treated with different concentrations of GSK-J4 (0, 2, 4, 6, 8, 10 μM) for 24, 48, 72 and 96 h, as for the effect of the decitabine on cell proliferation activity,KG-1a cells were also exposed to different concentrations of decitabine (0, 1, 5, 10 μM) for 24, 48 and 72 h. Also, the synergistic effect of GSK-J4 and decitabine on cell growth was assessed after KG-1a cells treated with 4 μM GSK-J4 and 5 μM decitabine for 24,48 and 72 h. Finally, 10 μl of CCK-8 solution was added into the cell culture medium. Optical density (OD) measurements were carried out after incubation for 2 h. The OD value of each well was recorded at 450 nm using a micro-plate absorbance reader.

### Cell cycle analysis

To assess the cell cycle distribution, 5 × 10^5^ cells were seeded into a 6-well plate. After the indicated treatment, KG-1a cells were harvested and washed twice with PBS. Then, the cells were fixed with 75% ethanol (absolute ethanol/PBS ratio = 3:1) at − 4 °C overnight. After incubation with propidium iodide (PI; 100 μg/ml) solution for 30 min at room temperature (RT) in the dark, the cells were measured using a CytoFLEX flow cytometer (Beckman Coulter, USA). The cell cycle distribution was analyzed with CytExpert V2.3.0.84 software (Beckman Coulter, USA).

### Cellular apoptosis detection

For the evaluation of cellular apoptosis, GSK-J4-treated cells were seeded into 6-well plates at a density of 5 × 10^5^ cells/well. After culturing for 48 h, the cells were harvested and washed twice with PBS. The rate of apoptotic cells was determined by the Annexin V-FITC Apoptosis Detection Kit (SUNGENE BIOTECH, Tianjing). Flow cytometric analysis was performed using a CytoFLEX flow cytometer (Beckman Coulter, USA). The apoptotic rate of PKC-α siRNA-transfected cells was evaluated using the same method.

### Western blot analysis

After the indicated treatments, the cells were collected and washed twice with PBS. Total protein was extracted using the fixture of RIPA lysis buffer (no. P0013; Beyotime, China) and phenyl methane sulfonyl fluoride (PMSF; 100:1; Cell Signaling Technology, USA). The concentration of total protein was determined by the BCA protein assay kit (P0012; Beyotime, China). Then, the protein extract was mixed with 5× loading buffer, heated for 5 min, separated with sodium dodecyl sulfate polyacrylamide gel electrophoresis and then transferred onto polyvinylidene fluoride membranes. After blocking with 5% skimmed milk solution, the blocked membranes were incubated with primary antibodies at 4 °C overnight. After washing twice with Tris-buffered saline containing Tween-20 (TBST), the membranes were incubated with secondary antibodies for 1 h at room temperature and then washed twice with TBST again. The protein signals were detected with an enhanced chemiluminescence (ECL) Ultra Western HRP Substrate kit (WBUL S0100; EMD Millipore, USA) using an ECL visualization system (GE Healthcare, USA). Primary antibodies against β-Actin (M01263-1) and p-Bcl2 (ab218123) were purchased from BOSTER Biological Technology (CA, USA) and Abcam (Cambridge, MA, USA), respectively. The antibodies against P21 (WL0362), CyclinD1 (WL01435a), CyclinA2 (WL02964), Bax (WL01637), Cleaved-caspase9 (WL01838), Cleaved-caspae12 (WL00735), GRP78 (WL03157), ATF4 (WL02330) and PKC-α (WL02234) were obtained from Wanleibio (Shenyang, China).

### Quantitative real-time polymerase chain reaction (qRT-PCR) assay

Total RNA was extracted using TRIzol reagent (Takara, Japan). cDNA was synthesized with the PrimeScript™ RT reagent Kit (Takara, Japan). qRT-PCR was performed on a CFX Connect™ real-time PCR operating system (Bio-Rad, USA) using the SYBR^®^ Premix Ex Taq™ II kit (Takara, Japan). Primers were synthesized at Sangon Biotech (Shanghai, China). The sequences of primers used for qRT-PCR are shown in Table 1.

**Table 1:** The primers used for qPCR assay

Gene	Forward/reverse	Primer Sequence(5′–3′)
PKC-α	F R	GTCCACAAGAGGTGCCATGAA AAGGTGGGGCTTCCGTAAGT
GAPDH	F R	CATCACGCCACAGTTTCC ATCATCAGCAATGCCTCC

### Statistical analysis

All statistical analyses were carried out using GraphPad Prism 5.0 and SPSS Statistics software (19.0). Statistical difference between two groups was compared using unpaired Student’s *t* test. The data were presented as mean ± standard deviation (SD). *p*-value < 0.05 was considered statistically significant.

## Results

### GSK-J4 induced cell growth inhibition and cell cycle arrest

**Fig. 1:**
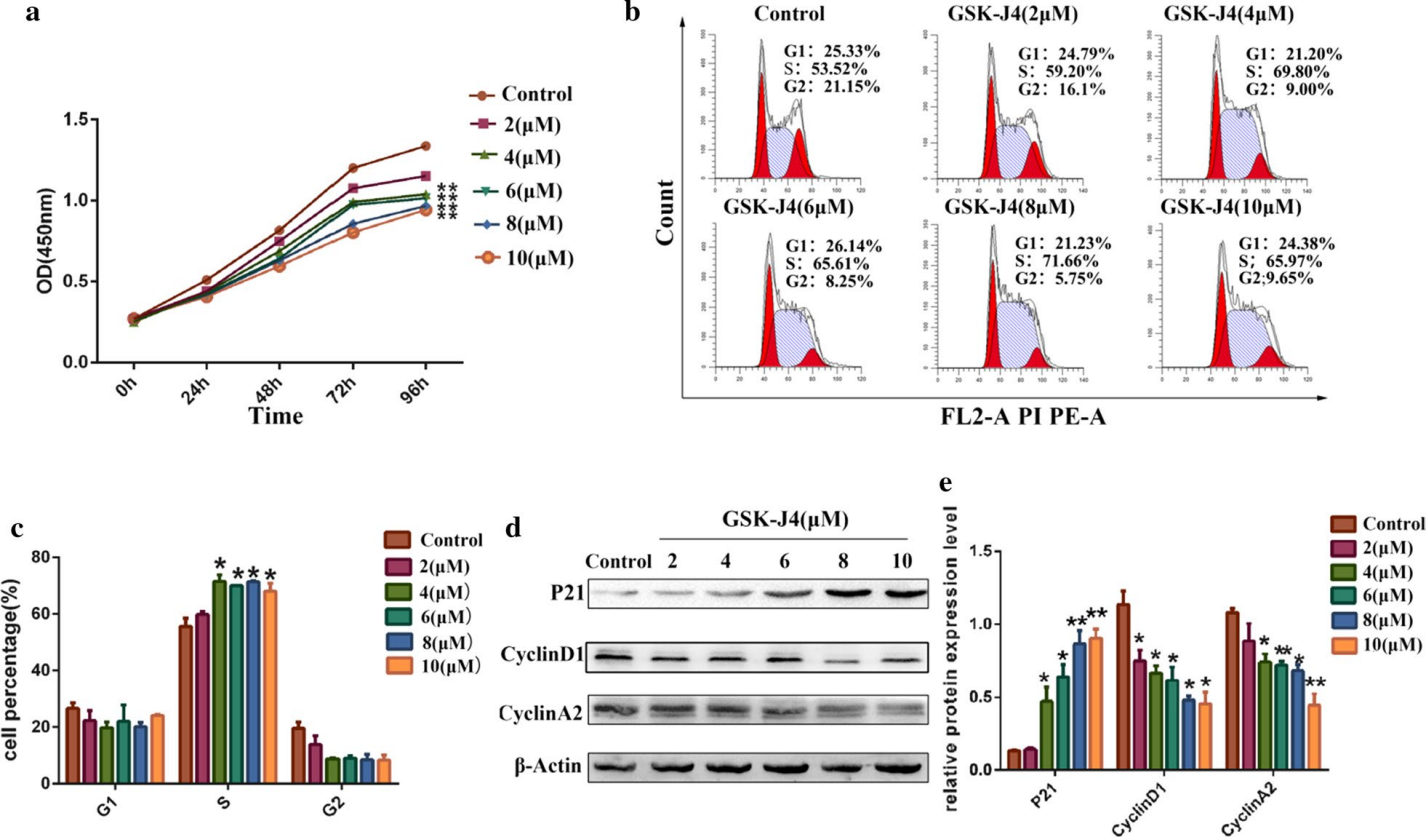
The effects of GSK-J4 on KG-1a cell proliferation and cell cycle distribution. **a** Cell viability was analyzed by the CCK-8 assay kit. **b** Cell cycle distribution was detected with flow cytometry. **c** The quantitative cell cycle distribution data. Values represent the mean ± SD of three independent experiments. *p < 0.05. **d** Western blotting was used to quantitatively analyze the expression levels of P21, CyclinD1 and CyclinA2. **e** Statistical analysis of the expression levels of P21, CyclinD1 and CyclinA2. β-Actin was used as an internal control. Values represent the mean ± SD of three independent experiments.*p < 0.05, **p < 0.01

Cell proliferation was monitored by using the CCK-8 assay. The CCK-8 data (Fig. 1a) showed that the viability of KG-1a cells was decreased in a dose-dependent manner after treatment with 2, 4, 6, 8 and 10 μM of GSK-J4 for 0, 24, 48, 72 and 96 h compared with the control group (p < 0.05). To examine the effect of GSK-J4 on cell growth inhibition, the distribution of KG-1a cell phase was evaluated by flow cytometric. As shown in Fig. 1a, b, GSK-J4 led to a notable accumulation of S phase cells in a dose-dependent manner (p < 0.05). After treatment with different concentrations of GSK-J4 for 48 h, the expression level of P21 was increased, while the expression levels of CyclinD1 and CyclinA2 were decreased significantly in a dose-dependent manner (p < 0.05) (Fig. 1d, e).

### GSK-J4 induces KG-1a cell apoptosis

**Fig. 2:**
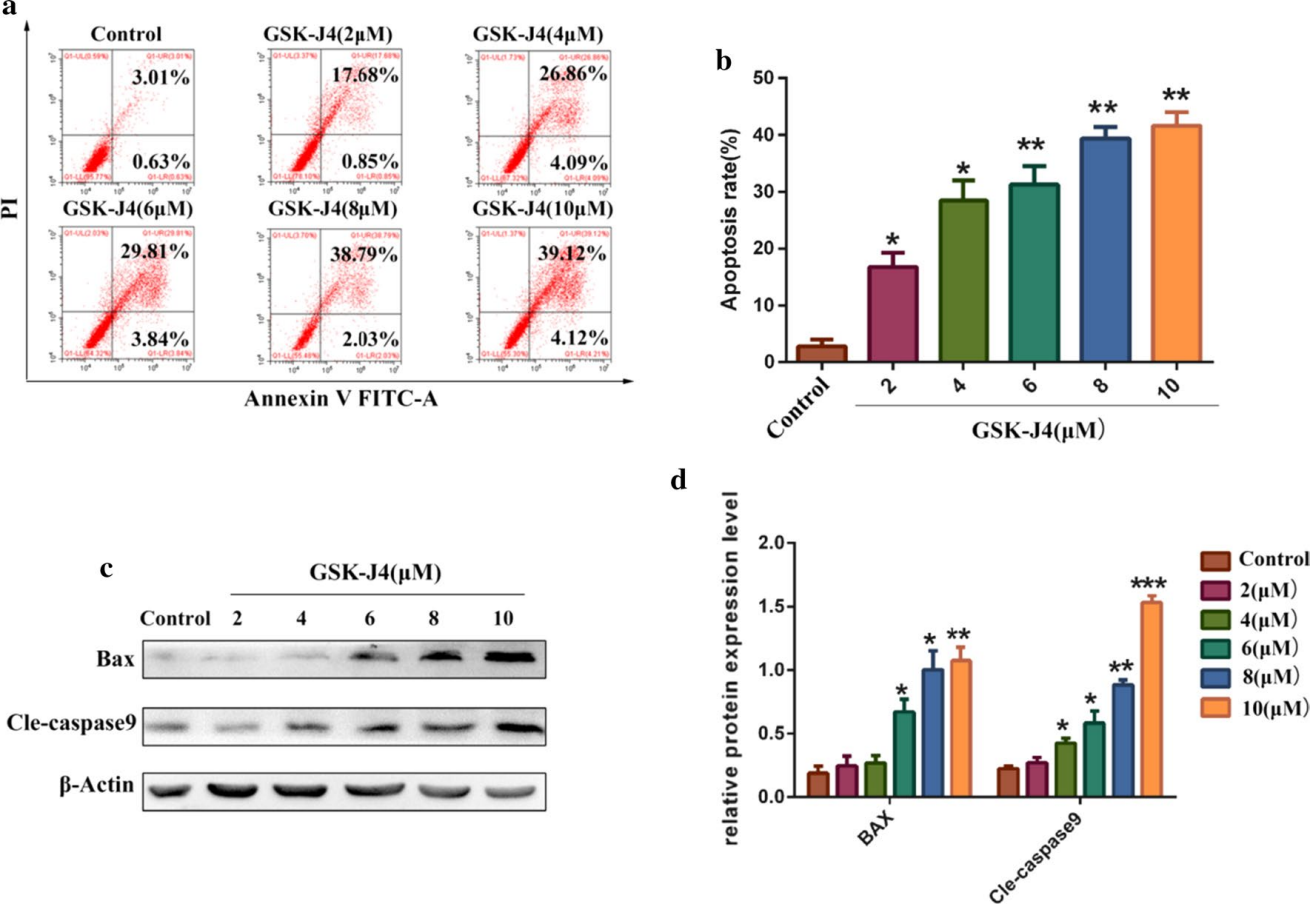
GSK-J4 induces KG-1a cell apoptosis. **a** The rate of cell apoptosis was detected by annexin-V and PI double-staining. **b** Statistical analysis of the apoptotic rate. Values represent the mean ± SD of three independent experiments.*p < 0.05, **p < 0.01. **c** Western blotting was used to analyze the expression levels of bax and cle-caspase9 in KG-1a cells after treatment with GSK-J4 for 48 h. **d** Statistical analysis of the expression levels of Bax and cle-caspase9. β-Actin was used as an internal control. Values represent the mean ± SD of three independent experiments.*p < 0.05, **p < 0.01, ***p < 0.001

To determine whether GSK-J4 can affect KG-1a cell apoptosis, several apoptotic parameters were assessed by flow cytometry and Western blotting. The flow cytometric data revealed that the apoptotic rate of KG-1a cells in GSK-J4 treatment group was significantly increased compared to the control group (p < 0.05)(Fig. 2a, b). Moreover, the results of Western blotting showed that the expression levels of apoptosis-related proteins (bax and cle-caspase9) were significantly increased in GSK-J4 treatment groups (p < 0.05) (Fig. 2c, d).

### GSK-J4 triggered ER stress

**Fig. 3:**
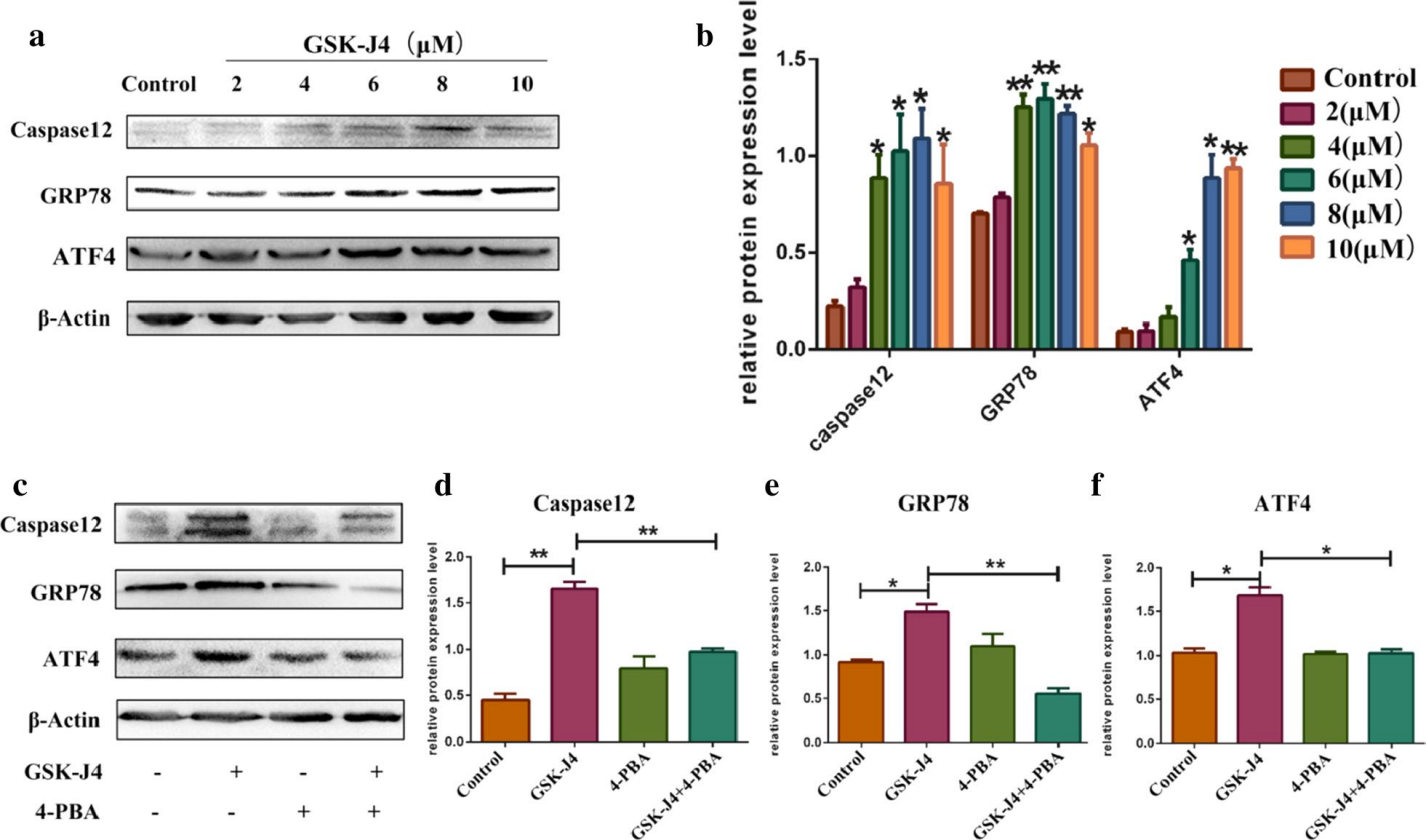
ER stress is stimulated by GSK-J4 in KG-1a cells. **a** The expression levels of caspase-12, GRP78 and ATF4 were detected by Western blotting. **b** Statistical analysis of the expression levels of caspase-12, GRP78 and ATF4. β-Actin was used as an internal control. Values represent the mean ± SD of three independent experiments.*p < 0.05, **p < 0.01. **c** After co-treatment with 4-PBA (3 mM) and GSK-J4 (4 μM), the expression levels of caspase-12, GRP78 and ATF4 in KG-1a cells were detected by Western blotting. **d**–**f** Statistical analysis of the expression levels of caspase-12, GRP78 and ATF4. β-Actin was used as an internal control Values represent the mean ± SD of three independent experiments.. *p < 0.05, **p < 0.01

To examine whether GSK-J4 can trigger ER stress, the protein expression levels of ER stress-related molecules, such as caspase-12, GRP78 and ATF4, were detected by Western blotting. As is shown in Fig. 3a, b. The protein levels of caspase-12, GRP78 and ATF4 were increased significantly in KG-1a cells treated with GSK-J4 compared to the control group (p < 0.05). To further confirm that GSK-J4 can stimulate ER stress, we detected the molecular indicators of ER stress in KG-1a cells after co-treatment with 4-phenyl butyric acid (4-PBA, the inhibitor of ER stress). The results of Western blotting indicated that the protein levels of caspase-12, GRP78 and ATF4 were remarkably lower in GSK-J4 (4 μM) and 4-PBA (3 mM) co-treatment group than those in GSK-J4 treatment alone group (p < 0.05) (Fig. 3c–f).

### ER stress was involved in GSK-J4 induced cell cycle arrest

**Fig. 4:**
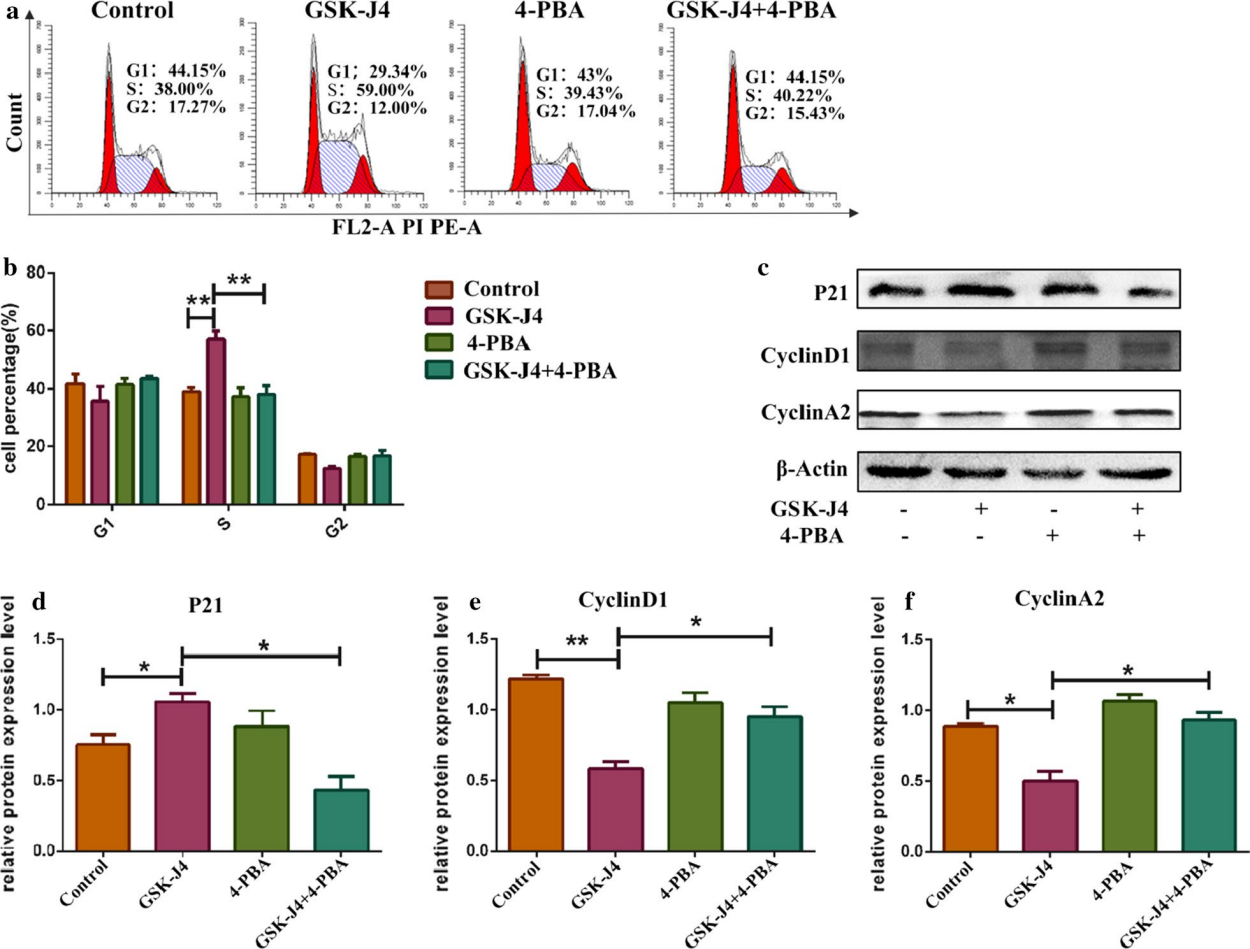
GSK-J4 induces cell cycle arrest via ER stress. **a** Pre-treatment with 4-PBA (3 mM) can alleviate the accumulation of cells in the S phase. **b** The quantitative cell cycle distribution data. Values represent the mean ± SD of three independent experiments. **p < 0.01. **c**. After pre-treatment with 4-PBA (3 mM), the expression levels of P21, CyclinD1 and CyclinA2 in KG-1a cells were detected by Western blotting. **d**–**f** Statistical analysis of the expression levels of P21, CyclinD1 and CyclinA2 in KG-1a cells exposed to 4-PBA, GSK-J4 and their combination. β-Actin was used as an internal control. Values represent the mean ± SD of three independent experiments.*p < 0.05, **p < 0.01 compared to the control group

To assess whether GSK-J4-induced cell cycle arrest is influenced by ER stress, KG-1a cells were pretreated with 3 mM 4-PBA and then treated with 4 μM GSK-J4 for 48 h. As shown in Fig. 4a, b, co-treatment with 4-PBA and GSK-J4 could alleviate the accumulation of KG-1a cells in the S phase (p < 0.05). Similarly, after co-treatment with 4-PBA, the expression level of P21 was downregulated while the expression levels of CyclinD1 and CyclinA2 were upregulated compared to GSK-J4 treatment alone group (p < 0.05) (Fig. 4c–e). These data suggest that ER stress is involved in the process of GSK-J4-induced cell cycle arrest.

### ER stress was involved in GSK-J4 induced cell apoptosis

**Fig. 5:**
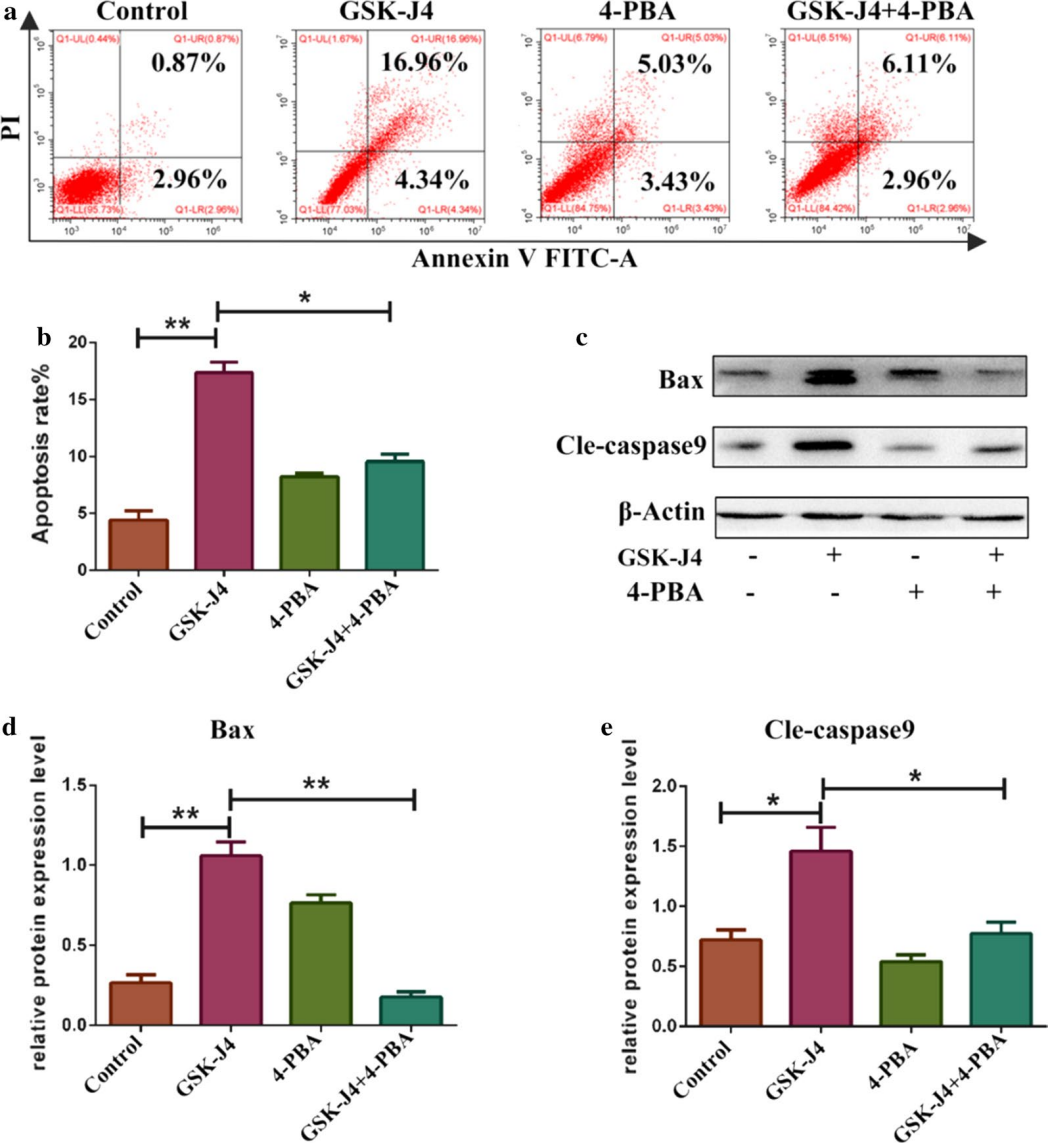
GSK-J4 induces cell apoptosis via ER stress. **a** After co-treatment with 4-PBA (3 mM) and GSK-J4 (4 μM), the ratio of apoptotic cells was analyzed using flow cytometry. **b** Statistical analysis of the apoptotic rate. Values represent the mean ± SD of three independent experiments.*p < 0.05, **p < 0.01. **c** After pre-treatment with 3 mM 4-PBA, the expression levels of bax and cleaved-caspase9 were detected by Western blotting. **d**, **e** Statistical analysis of the expression levels of Bax and cle-caspase9. β-Actin was used as an internal control. Values represent the mean ± SD of three independent experiments. *p < 0.05, **p < 0.01

To verify whether GSK-J4-induced cell apoptosis is mediated by ER stress, KG-1a cells were pretreated with 3 mM 4-PBA and then treated with GSK-J4 for 48 h. As shown in Fig. 5a, b, the co-treatment with 4-PBA could reduce the apoptotic rates of KG-1a cells (p < 0.05). After co-treatment with 4-PBA, the expression levels of bax and cle-caspase9 were decreased compared to GSK-J4 treatment alone group (p < 0.05) (Fig. 5c, d). These data suggested that ER stress is also involved in the process of GSK-J4-induced cell apoptosis.

### GSK-J4 inhibited PKC-α/p-Bcl2 pathway via ER stress

**Fig. 6:**
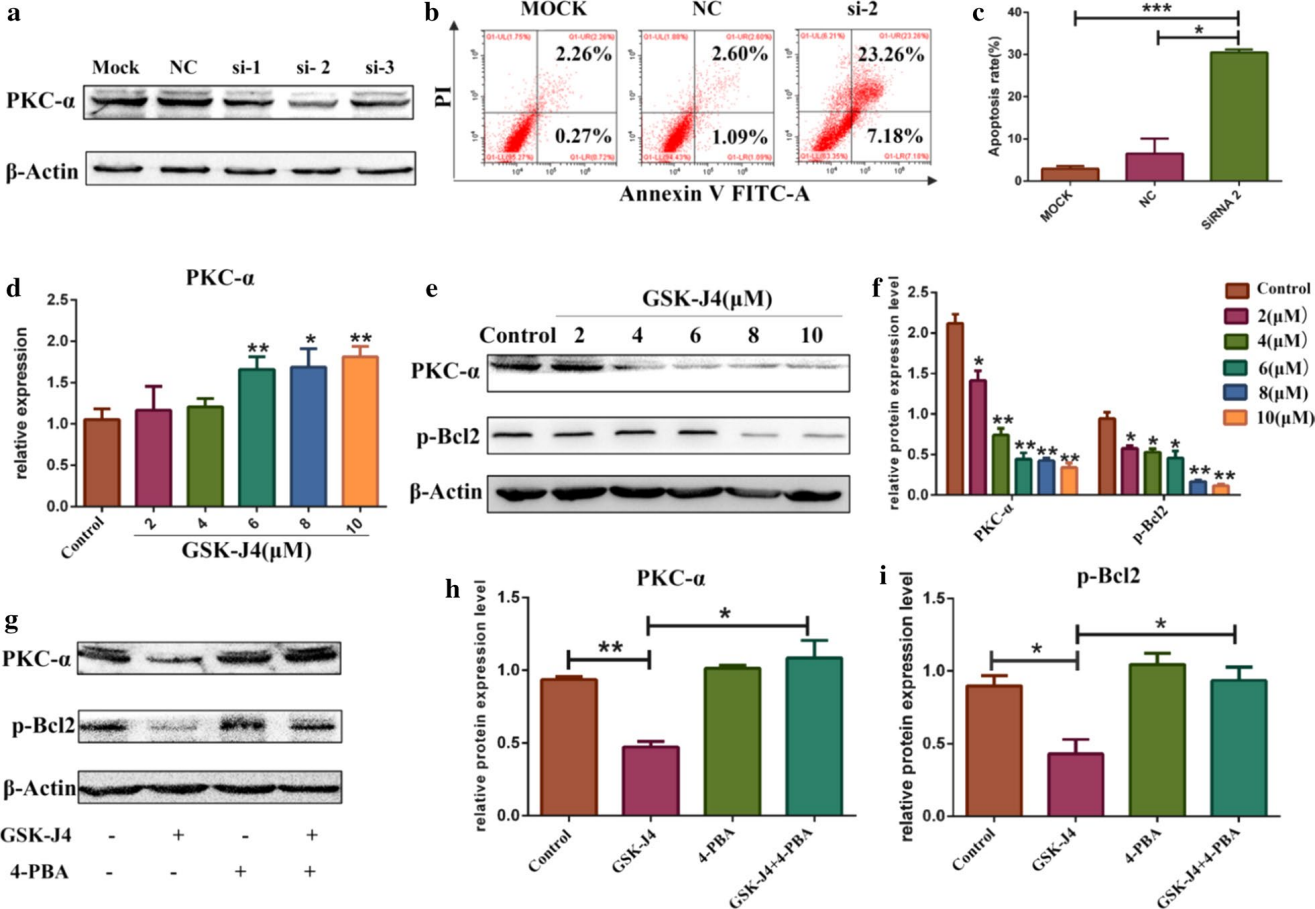
The role of ER stress in GSK-J4-regulated PKC-α/p-Bcl2 pathway inhibition. **a** After treatment with PKC-α siRNA NC, siRNA1, siRNA2 and siRNA3, the expression level of PKC-α was detected by Western blotting. **b** After treated with siRNA2, the ratio of apoptotic cells was detected using flow cytometry. **c** Statistical analysis of the apoptotic rate of KG-1a treated with siRNA2. Values represent the mean ± SD of three independent experiments. *p < 0.05, ***p < 0.001. **d** The mRNA expression level of PKC-α was detected using qRT-PCR. GAPDH was used as an internal control. Values represent the mean ± SD of three independent experiments. *p < 0.05, **p < 0.01. **e** After treatment with GSK-J4, the expression levels of PKC-α and p-Bcl2 in KG-1a cells were detected by Western blotting. **f** Statistical analysis of the expression levels of PKC-α and p-Bcl2. β-Actin was used as an internal control. Values represent the mean ± SD of three independent experiments. *p < 0.05, **p < 0.01. **g** After pre-treatment with 4-PBA (3 mM), the expression levels of PKC-α and p-Bcl2 were detected by Western blotting. **h**, **i** Statistical analysis of the expression levels of PKC-α and p-Bcl2. β-Actin was used as an internal control. Values represent the mean ± SD of three independent experiments. *p < 0.05, **p < 0.01

Further, we examined whether PKC-α/p-Bcl2 pathway inhibition is involved in the process of GSK-J4-induced apoptosis. Firstly, we investigated the anti-apoptotic role of PKC-α in AML. KG-1a cells were treated with siRNA NC, siRNA1, siRNA2 and siRNA3, the knock-down effects were then assessed by Western blotting (Fig. 6a).The knock-down efficiency of siRNA2 was found to be the most significant. Hence, we treated KG-1a cells with siRNA2 and detected the rate of apoptotic cells. As shown in Fig. 6b, c, when PKC-α was knocked-down, the apoptotic rates of KG-1a cells were increased significantly (p < 0.05). Next, we studied whether GSK-J4 can regulate the expression of PKC-α. KG-1a cells were treated with different concentrations of GSK-J4. As shown in Fig. 6d, the mRNA expression levels of PKC-α were significant higher in 6, 8 and 10 μM GSK-J4 treatment groups than those in control group (p < 0.05). Given that PKC-α could regulate the phosphorylation of Bcl2 (p-Bcl2) to inhibit cell apoptosis [41], the protein expression levels of PKC-α and p-Bcl2 were evaluated in KG-1a cells treated with GSK-J4. Notably, GSK-J4 treatment downregulated the protein expression levels of PKC-α and p-Bcl2 (p < 0.05) (Fig. 6e, f). To determine whether GSK-J4 can inhibit PKC-α/p-Bcl2 pathway by inducing ER stress, we detected the expression levels of PKC-α and p-Bcl2 in KG-1a cells treated with 4-PBA (3 mM) and GSK-J4(4 μM). Interestingly, the expression levels of PKC-α and p-Bcl2 were significantly lower in 4-PBA and GSK-J4 co-treatment group compared to GSK-J4 treatment alone group (p < 0.05) (Fig. 6g–i).

### The combination effects of decitabine and GSK-J4 in KG-1a cells

**Fig. 7:**
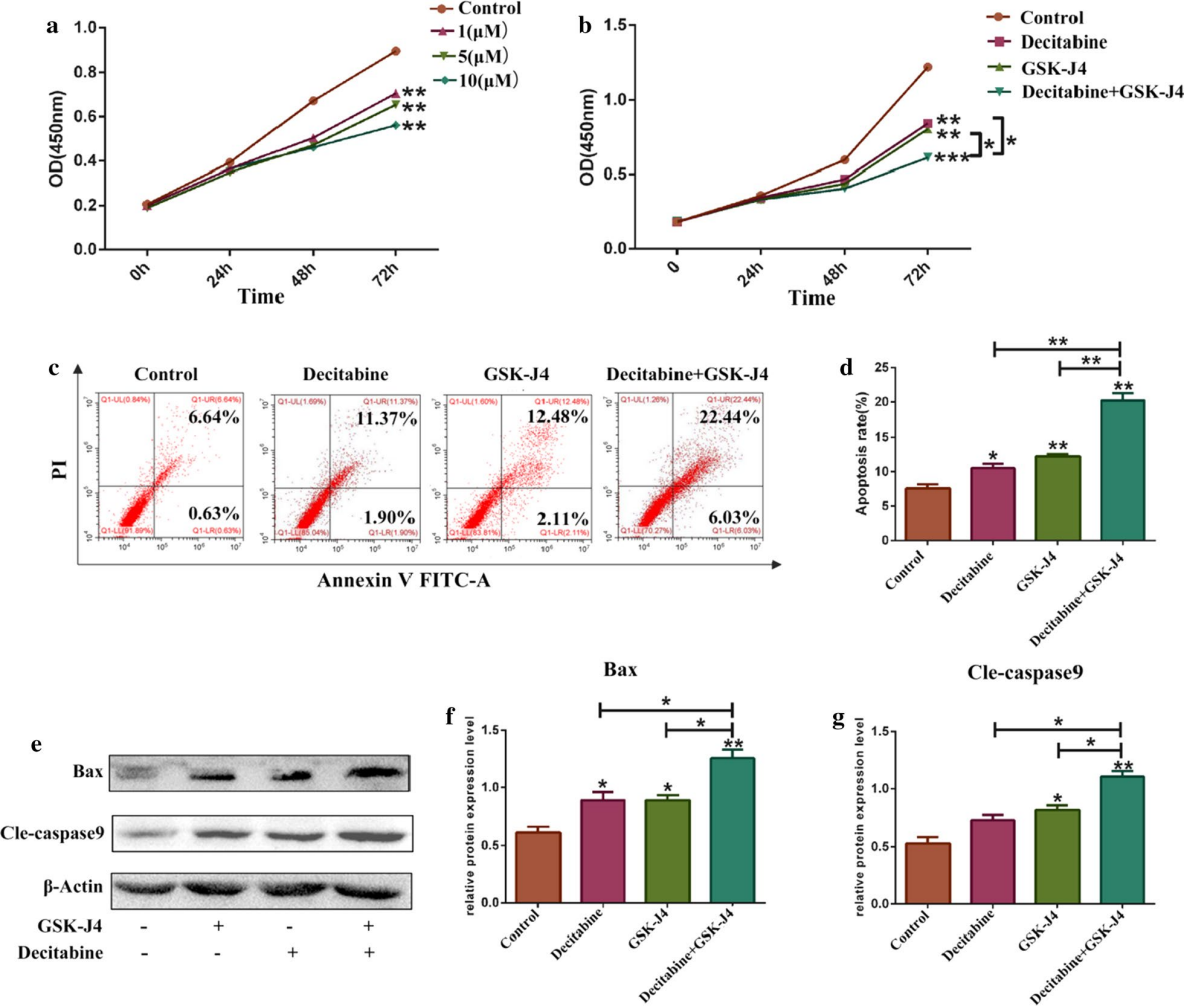
GSK-J4 exerts a synergistic effect with decitabine in KG-1a cells. **a** Cell viability was analyzed using the CCK-8 assay kit to detect the effect of decitabine on KG-1a cell proliferation. **b** Cell viability was also detected with CCK-8 assay kit to evaluate the combination effect of decitabine and GSK-J4 on KG-1a cell growth. **c** Following exposure to GSK-J4 and decitabine, the ratio of apoptotic cells was analyzed using flow cytometry. **d** Statistical analysis of the apoptotic rate.Values represent the mean ± SD of three independent experiments. *p < 0.05, **p < 0.01. **e** The expression levels of bax and cle-caspase-9 in KG-1a cells exposed to decitabine and GSK-J4 were detected by Western blotting. **f**, **g** Statistical analysis of the expression levels of Bax and cle-caspase-9. β-Actin was used as an internal control. Values represent the mean ± SD of three independent experiments. *p < 0.05, **p < 0.01

To explore whether the combination of decitabine and GSK-J4 can exert better outcomes in KG-1a cells, we detected both cell proliferation and expression levels of cell apoptosis-related indicators. As shown in Fig. 7a, decitabine could inhibit the growth of KG-1a cells after treatment with 1, 5 and 10 μM of decitabine for 0, 24, 48 and 72 h when compared to the control group (p < 0.05). After treatment with decitabine (5 μM) and GSK-J4 (4 μM), the viability of KG-1a cells was significantly inhibited in the combined treatment group compared to decitabine or GSK-J4 treatment alone group (p < 0.05) (Fig. 7b).This suggested that decitabine and GSK-J4 exhibit synergistic effects on the inhibition of KG-1a cell growth. To further investigate the effect of this combination on KG-1a cell apoptosis, the apoptotic index values were evaluated by flow cytometry and Western blotting. As shown in Fig. 7c, d, the apoptotic rate of KG-1a cells was increased markedly in the combined treatment group compared to decitabine or GSK-J4 treatment alone group (p < 0.05). Furthermore, the expression levels of Bax and cle-caspase-9 were significantly higher in the combination therapy group than those in monotherapy groups (p < 0.05) (Fig. 7e–g).

## Discussion

The hallmarks of AML cells are aberrant cell cycle and apoptosis dysregulation, which lead to poor clinical outcomes, such as low survival rates, poor prognosis, adverse drug reactions, and even drug resistance in AML patients treated with chemotherapy [26]. Growing evidence has suggested that GSK-J4 can be effectively used to treat blood disorders, including acute lymphoblastic leukemia and diffuse large B-cell lymphoma [27, 28]. However, the role and underlying mechanism of GSK-J4 in AML have not been fully explored. In this study, KG-1a cells were selected as the experimental model for studying the effects of GSK-J4 on AML cell growth, apoptosis and cell cycle distribution. Our findings demonstrated that GSK-J4 upregulated the expression of ER stress-related signaling molecules, such as GRP78, ATF4 and caspase-12, which are closely associated with cell cycle distribution and apoptosis regulation [29–32]. This inspires us to explore the role of ER stress in GSK-J4 function.

ER stress is an important biological reaction. When exposed to different types of stimulating factors, GRP78 would be separated from ER transmembrane proteins such as inositol-requiring enzyme 1, activating transcription factor 6 and RNA-dependent protein kinase-like ER kinase [33]. Those activated proteins then initiated unfolded protein response to maintain cellular homeostasis, thus protecting the cells from damage. Hence, GRP78 can be regarded as the marker of ER stress. Yet, the severe and persistent stimulation could trigger ER stress and then begin to affect many biological processions, such as cell growth, cell cycle, cell apoptosis and so on. P21 is an important cell cycle regulatory molecule that can be mediated by ER stress, and it may inhibit the cells from S phase into G2 phase by decreasing the expression levels of CyclinD1 and CyclinA2 [34–36]. In the present study, we confirmed that GSK-J4 treatment could induce cell cycle arrest at the S phase by downregulating the expression of CyclinD1 and CyclinA2 and upregulating that of P21. Caspase-12 is located on ER transmembrane, which can be activated by ER stress and plays a vital role in ER stress-related cell apoptosis. However, the proapoptotic role of caspase-12 has not been explained completely. A previous study has indicated that caspase-12 can activate other Caspase family members, including caspase-9 [37]. From this study, it was noted that GSK-J4 could upregulate the expression of cle-caspase-9 and another apoptosis index (bax), and the rate of apoptotic cells was increased as well. However, such effects of GSK-J4 could be markedly reversed by ER stress inhibitor 4-PBA, implying that GSK-J4 affects cell cycle distribution and apoptosis by inducing ER stress. These results further proved the crucial role of ER stress in treating AML.

Recent studies have established that the antiapoptotic role of PKC-α in AML, and its association with poor prognosis and chemoresistance [38, 39]. In this study, we also proved the antiapoptotic role of PKC-α by silencing PKC-α. A previous research has pointed out that GSK-J4 may exhibit other biological effects on cancer cells, apart from regulating methylation level [40]. Our results demonstrated that GSK-J4 could indeed decrease the expression of PKC-α and GSK-J4 treatment might affect PKC-α expression by regulating its protein level instead of mRNA level. This is probably attributed to the different cell lines employed and GSK-J4 may regulate the expression of PKC-α via post-transcriptional regulation, which implicated that GSK-J4 could not be only used to regulate H3K27 demethylation in biological processes, and next we need to explore the underlying mechanism of GSK-4 in regulating PKC-α expression. In fact, the antiapoptotic function of PKC-α has been shown to be mediated by the phosphorylation of Bcl2 to promote cell survival, inhibit cell apoptosis and mediate drug resistance in leukemia [41–43]. From the experimental results, it can be seen that GSK-J4 reduced the expression levels of PKC-α and pBcl2 and this effect was inhibited by 4-PBA. Therefore, PKC-α/pBcl2 pathway may participate in ER stress-related apoptosis induced by GSK-J4, and these results serve to further elucidate the apoptosis-inducing mechanism of GSK-J4. Several studies have suggested that PKC-α can regulate ER stress [44, 45], while the effect of ER stress on the expression level of PKC-α has not been studied. Here, we confirmed that ER stress could regulate PKC-α/pBcl2 pathway to stimulate apoptosis.

Given that ER stress inducer can exert better anti-leukemic effects by combining with other chemotherapeutic drugs, GSK-J4 is speculated to be beneficial when used in combination with conventional drugs for AML treatment. Decitabine is the strongest inhibitor of DNA methylation, which is often used to cure myelodysplastic syndrome and AML [46]. Decitabine can be used alone in AML, however, it usually associated with low complete response rate and poor survival when AML patients are treated with decitabine alone [47]. Recently, researchers have found that the co-treatment of decitabine with other drugs can exhibit much better outcomes in AML patients compared to decitabine treatment alone, especially in elderly AML patients [48]. Likewise, when GSK-J4 is used in combination with other chemotherapy drugs, it may exert a strong synergistic effect to overcome the drug resistance in cancer cells [49]. KG-1a cells are not sensitive to decitabine compared to other AML cell lines [50]. Hence, we studied whether GSK-J4 can enhance the pro-apoptotic effect of decitabine on KG-1a cells. To our expectation, the findings revealed that the proportion of viable cells was decreased significantly and that of apoptotic cells was increased markedly after co-treatment with GSK-J4 and decitabine compared to GSK-J4 or decitabine treatment alone, which confirmed the beneficial effects of this combined treatment. These results imply that the combination of decitabine and GSK-J4 is feasible in curing AML, and this combination could be used transiently in the clinic.

It is worth noting that KG-1a cells are often used for the study of leukemic stem cells, because KG-1a cells are restricted within the CD34 (+) CD38 (−) as similar to the malignant leukemic stem cells which could mediate the relapse of AML [51]. Besides, KG-1a cells exhibit a high level of resistance to many clinical drugs, owning to the high capacity of p-glycoprotein-mediated drug efflux [52]. The noticeable roles of GSK-J4 in inhibiting KG-1a cell viability and inducing cell apoptosis confirmed that GSK-J4 may be helpful for the treatment of drug-resistant and relapsed AML patients. Therefore, it is necessary to investigate whether GSK-J4 can be used to overcome drug resistance in AML cells. GSK-J4 exhibits less toxicity to normal cells and has an effective membrane permeability [2, 53], which make it possible to act as a promising drug for AML treatment. Hence, we need to explore whether GSK-J4 can induce toxicity to normal hematopoietic stem cells in the future work. In addition, promising results were reported in high-risk neuroblastoma treated with the combination of both GSK-J4 and venetoclax (bcl-2 inhibitor) [20].However, we also know little about the cooperation therapeutic effect of GSK-J4 and venetoclax in AML especially in primary refractory/relapsed AML. Further research may include both GSK-J4 and venetoclax to study the potential treatment effects.

This research, however, is subjected to several limitations which should be considered. The first one is that only one AML cell line was included in our study, maybe it is not enough to prove that GSK-J4 has the same effects on all AML cell lines. Other AML cell lines should also need to be used to investigate the role of GSK-J4. The second limitation concerns is that we didn’t figure out the inner mechanism of GSK-J4 regulating the expression of PKC-α. Equally important is the animal experiment. All the results were based on cell experiments, nevertheless, the effect of GSK-J4 and ER stress inhibitor is not clear and needs to be studied in vivo. We will explore the combination effects of GSK-J4 and decitabine on tumor inhibition in vivo in follow-up experiments too.

## Conclusion

**Fig. 8:**
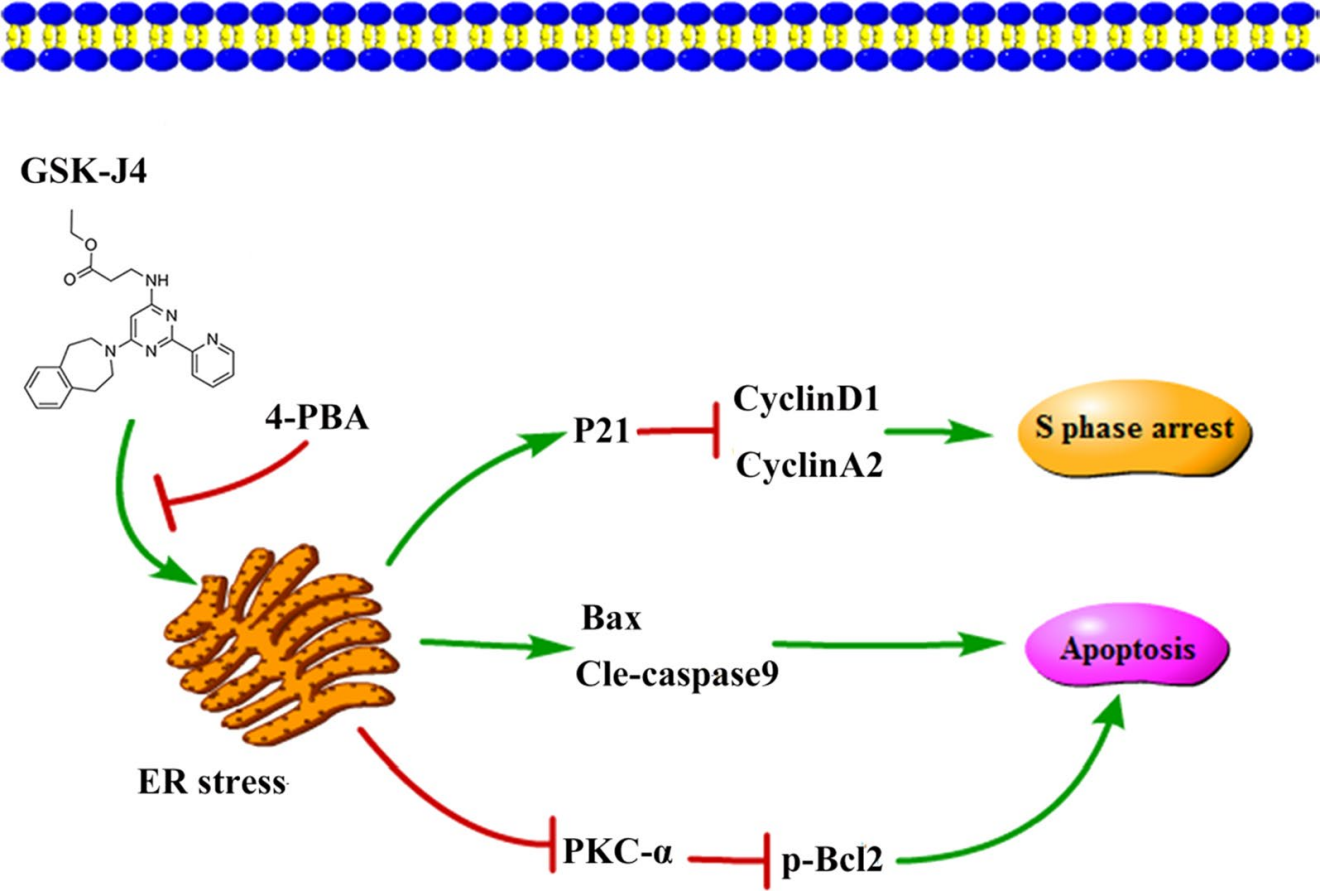
The mechanisms of GSK-J4-induced cell cycle arrest, cell apoptosis and PKC-α/p-Bcl2 pathway inhibition. GSK-J4 induces S phase arrest and cell apoptosis through ER stress, and inhibits PKC-α/p-Bcl2 pathway by stimulating ER stress

In summary, our study reveals that GSK-J4 induces KG-1a cell apoptosis and cell cycle arrest at the S phase via triggering ER stress. In addition, the antiapoptotic role of PKC-α/p-Bcl2 pathway in KG-1a cells is inhibited by ER stress after treatment with GSK-J4, as illustrated in the schematic diagram (Fig. 8). Furthermore, our findings indicate that the combination of decitabine and GSK-J4 can serve as a promising therapeutic strategy for AML patients who are insensitive to decitabine.

## Data Availability

All data analyzed and generated during the current study are available from the corresponding author upon reasonable request.

## Abbreviations

AML: Acute myeloid leukemia
4-PBA: 4-Phenyl butyric acid
ER: Endoplasmic reticulum
UPR: Unfolded protein response
